# Hypertensive disorders of pregnancy increase neonatal cerebral regional tissue oxygen saturation in the early postnatal period: partial mediation by preterm birth

**DOI:** 10.3389/fcell.2025.1682675

**Published:** 2025-11-27

**Authors:** Yao Zhang, Tong Yang, Dengjun Liu, Jiaxi Wu, Yanxia Mao, Dapeng Chen, Jun Tang, Yi Yang, Tao Xiong

**Affiliations:** 1 Department of Pediatrics, West China Second University Hospital, Sichuan University, Chengdu, China; 2 Key Laboratory of Birth Defects and Related Diseases of Women and Children (Sichuan University), Ministry of Education, Chengdu, China; 3 Department of Pediatric Otolaryngology Head and Neck Surgery, West China Second University Hospital, Sichuan University, Chengdu, China

**Keywords:** hypertensive disorders of pregnancy, placental insufficiency, neonate, cerebral oxygenation, preterm birth, mediation effect

## Abstract

**Objective:**

Hypertensive disorders of pregnancy (HDP) are closely related to placental disfunction and may association neonatal cerebral oxygenation. This study aimed to assess the correlation of maternal HDP on neonatal cerebral regional tissue oxygen saturation (crSO_2_) and cerebral fractional tissue oxygen extraction (cFTOE), and to determine whether preterm birth mediates these associations.

**Methods:**

An observational case-control study enrolled 16799 newborns as the basis for subsequent screening. Finally, 464 infants born to mothers exposed to HDP were selected as the case group, and 464 normal mothers were selected as the control group. Cerebral oxygenation was monitored weekly using near-infrared spectroscopy from birth until 28 days postnatally or discharge. crSO_2_ values were recorded, and cFTOE was calculated at each time point. Generalized linear mixed models were employed to evaluate the association of HDP (and the subset of preeclampsia) with serial crSO_2_ and cFTOE measurements. The mediation role of preterm birth was assessed using Model four of the SPSS PROCESS macro.

**Results:**

Neonates exposed to maternal HDP/preeclampsia showed significantly higher crSO_2_ (β = 0.472, 95% CI: 0.122–0.823, p = 0.008; β = 0.625, 95% CI: 0.234–1.017, p = 0.002, respectively) and lower cFTOE (β = −0.004, 95% CI: −0.008 to −0.001, p = 0.027; β = −0.006, 95% CI: −0.010 to −0.002, p = 0.006, respectively) during the first postnatal week compared to controls. Mediation analysis indicated that preterm birth accounted for 20.34% of the association between HDP and crSO_2_, and 17.85% of the association between preeclampsia and crSO_2_.

**Conclusion:**

Maternal HDP is associated with elevated neonatal cerebral oxygenation and reduced cerebral oxygen extraction in the early postnatal period. These associations are partially mediated by preterm birth, which may be linked to impaired placental function in pregnancies complicated by HDP.

## 1 Introduction

Hypertensive disorders of pregnancy (HDP) encompass a spectrum of conditions that frequently complicate gestation and contribute substantially to maternal and neonatal morbidity (Vesoulis et al., 2021a). These disorders are often characterized by abnormal placental development and vascular dysfunction, which may compromise intrauterine oxygen and nutrient delivery (Liu et al., 2022). Such intrauterine disturbances may adversely affect fetal brain development, even in the absence of overt structural anomalies at birth (Barron et al., 2021).

Epidemiological studies have shown that offspring exposed to HDP are at increased risk of neurodevelopmental disorders, such as intellectual disability, attention-deficit/hyperactivity disorder, and autism spectrum disorder (Wang et al., 2021; Maher et al., 2018; Sun et al., 2020; Atkinson et al., 2025). Although these disorders typically manifest in later childhood or adolescence, the critical windows of vulnerability during development remain undefined.

The fetal nervous system undergoes rapid structural and functional changes during gestation and requires a continuous supply of oxygen. This high metabolic demand might render the developing brain particularly vulnerable to injury associated with low peripheral arterial oxygen saturation when exposed to adverse intrauterine conditions (Katheria et al., 2021). In the context of HDP, maternal vascular abnormalities might be associated with placental dysfunction, resulting in a suboptimal intrauterine environment that may disrupt fetal cerebral development (Huang et al., 2022; Xiong et al., 2018; Liu et al., 2022; Wang et al., 2021; Maher et al., 2018; Brand et al., 2021). Therefore, assessing neurological function during the neonatal period in HDP-exposed infants helps identify those at risk and supports timely follow-up and intervention.

Near-infrared spectroscopy (NIRS) estimates regional cerebral oxygen saturation (crSO_2_) indirectly based on near-infrared light absorption spectra, assuming fixed optical and hemodynamic properties of brain tissue, rather than directly measuring oxygen saturation in the brain (Martini and Corvaglia, 2018). In neonates, anatomical features such as the thinner calvarial bones and a shorter scalp-to-cortex distance enhance signal accuracy. CrSO_2_ values measured by NIRS closely correlate with cerebral perfusion (Vesoulis et al., 2021a; Arman et al., 2020), making it a reliable tool for assessing oxygen delivery and consumption in neonatal brain (Ziehenberger et al., 2018). Deviations in these values can serve as early indicators of neurological dysfunction (Pichler et al., 2023).

Most neonatal NIRS studies have examined the correlation of clinical interventions on crSO_2_ (Pichler et al., 2023; Chock et al., 2023; Pellegrino et al., 2023; Sutin et al., 2023), while only a few have addressed the effects of maternal antihypertensive medication in HDP cases (Richter et al., 2020; Thewissen et al., 2017; Richter et al., 2016). These studies were generally limited to the first 5 days after birth, with little information on cerebral oxygenation beyond the early neonatal phase. However, in severe HDP, impaired fetal cerebral autoregulation may persist after birth and potentially lead to prolonged neurological vulnerability (Escudero et al., 2023). Therefore, it is important to clarify how long and through what mechanisms HDP may affect neonatal crSO_2_.

Furthermore, HDP is a major contributor to preterm birth (Calek et al., 2024; Nilsson et al., 2024), which is linked to adverse neurological outcomes, including severe intraventricular hemorrhage, particularly when accompanied by white matter injury, and impaired cerebral oxygenation (Bell et al., 2022; Mohamed et al., 2021; Logan et al., 2013; Vohr, 2022). However, it remains unclear whether the neurological abnormalities observed in HDP-exposed infants result directly from HDP or indirectly through HDP-induced prematurity (Escudero et al., 2023). We hypothesize that the neurological dysfunction observed in HDP-affected offspring at birth may be attributable to both HDP-related intrauterine placental perfusion insufficiency and preterm birth triggered by HDP.

To investigate the association and temporal pattern of HDP on neonatal crSO_2_, we conducted continuous NIRS monitoring throughout the neonatal period in infants born to mothers with HDP. We also performed mediation analysis to assess whether preterm birth, represented by gestational age (GA), mediates the association between HDP and crSO_2_. In addition, preeclampsia (PE), the most common subtype of HDP, was analyzed separately. This study aims to clarify the pathways through which HDP influences early brain function, providing insights that may inform clinical strategies for early identification and intervention in high-risk neonates.

## 2 Objectives and methods

### 2.1 Research object

This study included neonates admitted to the Department of Neonatology at West China Second University Hospital, Sichuan University, between January 2021 and March 2024. This case-control study included 464 infants born to mothers diagnosed with HDP. A total of 464 non-HDP newborns were randomly selected at a 1:1 ratio as the control group. Inclusion criteria were as follows: (1) complete maternal medical records; (2) singleton pregnancies; and (3) admission to the neonatal ward after birth available crSO_2_ monitoring data. Exclusion criteria were: (1) multiple pregnancies; (2) genetic or metabolic disorders; and (3) major congenital anomalies. This study was approved by the Ethics Committee of West China Second University Hospital (Approval Number: Z-2019-41-2101-04) and conducted in accordance with the STROBE (Strengthening the Reporting of Observational Studies in Epidemiology) guidelines.

### 2.2 Study design

#### 2.2.1 Diagnostic criteria for HDP

HDP were classified according to the diagnostic criteria of the American College of Obstetricians and Gynecologists (ACOG) into four categories: gestational hypertension, PE-eclampsia, chronic hypertension, and chronic hypertension with superimposed PE (ACoOa, 2013). PE was diagnosed by the onset of hypertension after 20 weeks of gestation, accompanied by proteinuria or disfunction of at least one organ or system. Proteinuria was defined as a 24-h urine protein level ≥0.3 g or urine protein-to-creatinine ratio ≥0.3.

#### 2.2.2 Data collection

Data were prospectively collected through standardized case report forms (CRFs) integrated into the Perinatal Medicine Electronic Information Platform at West China Second University Hospital.

Maternal data collection included: (1) Basic demographic and clinical information such as age, pre-delivery body mass index (BMI), BMI change during pregnancy, and intrapartum systolic and diastolic blood pressure; (2) Record of drugs exposure within 72 h before delivery, including the use and cumulative dose of nifedipine, urapidil, labetalol, and magnesium sulfate (MgSO_4_); (3) Pregnancy-related complications, defined according to the 10th revision of the International Classification of Diseases (ICD-10) and corresponding clinical guidelines. These include fetal distress, fetal growth restriction, obesity, advanced maternal age at first delivery, hypoproteinemia, thyroid dysfunction (hypothyroidism or hyperthyroidism), gestational diabetes, and intrahepatic cholestasis of pregnanc.

Neonatal data collection included: (1) Basic demographics characteristics recorded at birth, including sex, ethnicity, gravidity and parity, GA (weeks + days), birth weight (BW), body length, and Apgar scores at 1, 5, and 10 min; (2) Therapeutic interventions, such as mode and duration of mechanical ventilation, timing of the first blood transfusion, and cumulative transfusion volumes; (3) Clinical complications, categorized by organ systems including respiratory system (apnea, neonatal respiratory distress syndrome, bronchopulmonary dysplasia), circulatory system (patent ductus arteriosus), infectious diseases (all sepsis during the neonatal period is included), gastrointestinal system [gastrointestinal hemorrhage, necrotizing enterocolitis (Bell grade II or above)], neurological system (intraventricular hemorrhage and its grading, grade I and grade II intraventricular hemorrhage are defined as mild intraventricular hemorrhage, while grade III and grade IV intraventricular hemorrhage are defined as severe intraventricular hemorrhage), ophthalmologic conditions (retinopathy of prematurity and its staging), and growth-related conditions such as small-for-gestational-age status. All neonates were followed until discharge or up to postnatal day 28, whichever occurred first.

Maternal data were extracted from the obstetric inpatient electronic system and cross-checked against paper medical records by a designated researcher. Two independent reviewers verified the entered information to ensure consistency. Neonatal data were compiled daily by the NICU information system, with weekly quality audits conducted by the research team to maintain data accuracy and completeness.

#### 2.2.3 Outcomes

##### 2.2.3.1 crSO_2_

crSO_2_ was estimated using the EGOS-600B NIRS device (ENGINMED Co., China). Probes were placed on the mid-forehead region at rest and secured with elastic bandages to prevent light leakage (He et al., 2024). NIRS leverages the differential absorption characteristics of oxygenated hemoglobin (HbO_2_) and deoxygenated hemoglobin (Hb) in the near-infrared spectrum to determine regional tissue oxygen saturation, calculated as crSO_2_ = [HbO_2_/(HbO_2_ + Hb)] × 100%. Continuous monitoring was initiated after signal stabilization, and lasted for 20 min, with recordings taken at 5-min intervals. The average of five consecutive readings was used for analysis. Weekly assessments were conducted throughout hospitalization until either discharge or the 28th postnatal day, whichever occurred first.

##### 2.2.3.2 Fractional tissue oxygen extraction (cFTOE)

The cFTOE represents the balance between cerebral oxygen supply and consumption. It was calculated using the following formula: cFTOE = (SpO_2_ − crSO_2_)/SpO_2_, where SpO_2_ denotes peripheral arterial oxygen saturation estimated concurrently.

### 2.3 Statistical analysis

Data were analyzed using SPSS version 27.1 (IBM Corp., Armonk, NY, USA). Continuous variables with normal distribution were presented as mean ± standard deviation, and those with non-normal distribution were expressed as median and interquartile range. Differences among groups were assessed using analysis of t-test for normally distributed data, the Wilcoxon rank-sum test for non-normally distributed data, and chi-square tests for categorical data.

First, we examined the correlations among crSO_2_, cFTOE, HDP, and PE. Generalized linear mixed models were used to investigate the associations of HDP and PE with crSO_2_ and cFTOE collected at all time points. And the GA and BW were analyzed as correction factors. Due to incomplete data collection at the 3rd and 4th postnatal week follow-ups, the group-by-time interaction term was not included in the mixed model.

**FIGURE 1:**
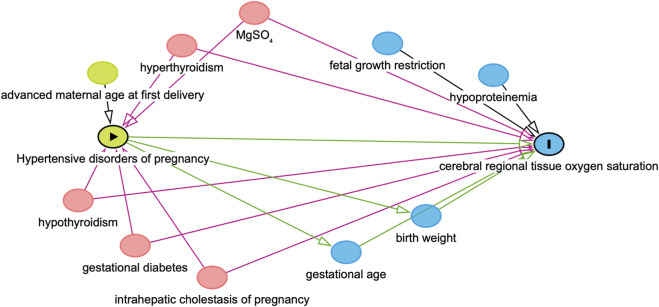
Directed acyclic graphs (DAGs). DAGs illustrating the variable selection process in this study. In this diagram, HDP serves as the exposure variable, while crSO_2_ is designated as the outcome variable. Covariates represented by green ovals are identified as common ancestors of the exposure, blue ovals denote common ancestors of the outcome, and pink ovals indicate common ancestors of both the exposure and the outcome.

Finally, the directed acyclic graph (DAG) was used to identify confounders along the backdoor paths between HDP and crSO_2_ and to avoid adjustment for mediators or downstream variables (Figure 1). Maternal factors (e.g., advanced maternal age at first delivery, hypoproteinemia, hypothyroidism or hyperthyroidism, gestational diabetes, intrahepatic cholestasis of pregnancy, and MgSO_4_ use) were included as confounders, while GA and BW were treated as physiological covariates. In the mediation analysis, fetal growth restriction was adjusted for as a potential confounder, whereas fetal distress was not included because it represents a downstream outcome of HDP. The mediating association of preterm birth was tested using Model four in the SPSS Process macro (Hayes and Rockwood, 2017). A p-value <0.05 was considered statistically significant.

## 3 Result

### 3.1 Baseline characteristics

**FIGURE 2:**
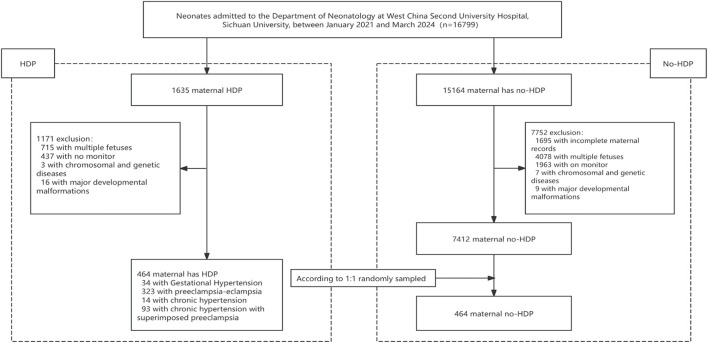
Flowchart of the study population screening process. This flowchart outlines the stepwise procedure for selecting the study participants. Initially, newborns admitted to the neonatal ward during the study period (January 2021 to March 2024) underwent eligibility assessment. Those born to mothers diagnosed with hypertensive disorders of pregnancy (HDP) were assigned to the HDP group. The control group was selected through a computer-generated randomization method with a 1:1 matching ratio from newborns whose mothers were not diagnosed with HDP. Individuals who failed to meet the inclusion criteria, had incomplete clinical records, or did not undergo cerebral tissue oxygen saturation monitoring were excluded from the final analysis.

A total of 928 neonates who met the inclusion criteria were enrolled in the study, including 464 born to mothers diagnosed with HDP and 464 randomly selected controls born to mothers without HDP during the same period (Figure 2). The median gestational age of the HDP group was 34.3 weeks, and the median birth weight was 1865 g. The median gestational age and birth weight of the non-HDP group were 34.6 weeks and 2200g, respectively. Among the HDP group, 323 infants (69.61%) were born to mothers with PE, 93 (20.04%) to mothers with chronic hypertension with superimposed PE, 34 (7.33%) with gestational hypertension, and 14 (3.02%) with chronic hypertension. Compared to the control group, mothers with HDP were older, had a higher proportion of advanced maternal age at first delivery, higher prenatal blood pressure, higher body mass index (BMI), increased incidence of fetal distress, fetal growth restriction, hypoproteinemia, hypothyroidism, gestational diabetes mellitus, intrahepatic cholestasis of pregnancy. They also received more frequent administration of antihypertensive medications, including nifedipine, urapidil, labetalol, and MgSO_4_ before delivery (Table 1). Neonates exposed to HDP had lower birth weights, fewer males, and fewer Han ethnicities (Table 2).

**TABLE 1:** Maternal baseline data.

Variables	No-HDP n = 464	HDP n = 464	*P*
Maternal age, year, M (Q_1_, Q_3_)	32.00 (29.00,35.00)	33.00 (30.00,36.00)	<0.001
Ederly primipara, (n, %)	45 (9.70)	77 (16.59)	0.002
Systolic blood pressure, mmHg, M (Q_1_, Q_3_)	117.00 (110.00,123.00)	140.50 (131.00,151.00)	<0.001
Diastolic pressure, mmHg, M (Q_1_, Q_3_)	70.00 (65.00,77.00)	90.50 (81.00,98.00)	<0.001
Body mass index, M (Q_1_, Q_3_)	25.11 (23.05,27.64)	27.34 (24.75,29.67)	<0.001
Fetal distress, (n, %)	2 (0.43)	11 (2.37)	0.001
Intrauterine growth restriction (n, %)	27 (9.41)	161 (34.70)	<0.001
Hypoproteinemia (n, %)	3 (0.65)	26 (5.60)	<0.001
Hypothyroidism (n, %)	67 (14.44)	92 (19.83)	0.029
Gestational diabetes (n, %)	148 (31.90)	186 (40.09)	0.009
Intrahepatic cholestasis of pregnancy (n, %)	27 (5.82)	49 (10.56)	0.008
Nifedipine			
72 h before delivery	22 (4.74)	121 (26.08)	<0.001
48 h before delivery	38 (8.19)	141 (30.39)	<0.001
24 h before delivery	53 (11.42)	178 (38.36)	<0.001
Urapidil			
72 h before delivery	0 (0.00)	7 (1.51)	0.019
48 h before delivery	0 (0.00)	15 (3.23)	<0.001
24 h before delivery	0 (0.00)	37 (7.99)	<0.001
Labetalol			
72 h before delivery	0 (0.00)	127 (27.43)	<0.001
48 h before delivery	0 (0.00)	162 (34.91)	<0.001
24 h before delivery	0 (0.00)	211 (45.47)	<0.001
Magnesium sulfate			
72 h before delivery	47 (10.13)	176 (38.01)	<0.001
48 h before delivery	59 (12.72)	212 (45.69)	<0.001
24 h before delivery	63 (13.58)	278 (59.91)	<0.001

**TABLE 2:** Descriptive characteristics for infants.

Variables	No-HDP n = 464	HDP n = 464	*P*
Gestational age, weeks, M (Q_1_, Q_3_)	34.60 (32.70,35.90)	34.30 (32.30,36.00)	0.706
Birth weight, g, M (Q_1_, Q_3_)	2200 (1750,2580)	1865 (1400,2290)	<0.001
Male (n, %)	272 (58.62)	214 (46.12)	<0.001
Han ethnicity (n, %)	445 (95.91)	417 (89.87)	<0.001
Apnea (n, %)	13 (2.80)	22 (4.74)	0.121
Sepsis (n, %)	48 (10.34)	38 (8.19)	0.258
Respiratory distress syndrome (n, %)	157 (33.84)	162 (34.91)	0.73
Bronchopulmonary dysplasia (n, %)	27 (5.82)	25 (5.39)	0.775
Retinopathy of prematurity (n, %)	32 (6.90)	31 (6.68)	0.896
Patent ductus arteriosus (n, %)	187 (40.30)	171 (36.85)	0.281
Intraventricular haemorrhage (n, %)			0.553
mild intraventricular hemorrhage (n, %)	52 (11.21)	42 (9.05)	
severe intraventricular hemorrhage (n, %)	7 (1.51)	7 (1.51)	
Non-invasive ventilator, n (%)	253 (54.53)	247 (53.23)	0.742
Invasive ventilator, n (%)	49 (10.56)	61 (13.15)	0.223
Blood transfusion, n (%)	28 (6.03)	33 (7.11)	0.508

### 3.2 Intergroup differences in crSO_2_ and cFTOE at different time point

**FIGURE 3:**
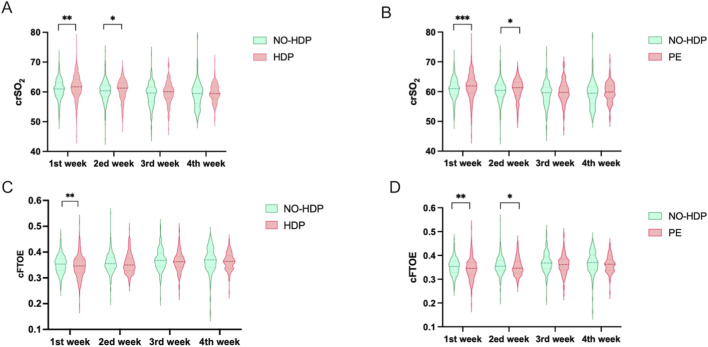
Comparative analysis of crSO2 and cFTOE across different time points among study groups. The t-test for normally distributed data, the Wilcoxon rank-sum test f or non-normally distributed data. *, p < 0.05; **, p < 0.01; ***, p < 0.001. **(A)** Comparison of crSO_2_ between the no-HDP group and the HDP group across all time points, along with intergroup differences; **(B)** Comparison of crSO_2_ between the no-HDP group and the PE group at each time point and corresponding intergroup differences; **(C)** Comparison of cFTOE between the no-HDP group and the HDP group at each time point, including intergroup differences; **(D)** Comparison of cFTOE between the non-HDP group and the PE group across all time points, together with intergroup differences.

Comparison with the control group, neonates born to mothers with HDP exhibited higher crSO_2_ levels during the first and second weeks. A similar pattern was observed in the PE subgroup, where neonates born to mothers with PE had higher crSO_2_ value at those time points (Figures 3A,B).

In contrast, neonates in the HDP group had lower cFTOE values during the first week compared with controls. This trend was more pronounced in the PE subgroup, where significantly lower cFTOE levels were observed during both the first and second weeks (Figures 3C,D).

### 3.3 Generalized linear mixed model analysis

In the unadjusted generalized linear mixed model analysis, both HDP and PE were associated with higher crSO_2_ levels compared to the control group (β = 0.520, 95% confidence interval (CI): 0.194 to 0.846, p = 0.002; β = 0.663, 95% CI: 0.305 to 1.022, p < 0.001, respectively), as well as with lower cFTOE values (β = - 0.005, 95% CI: 0.009 to - 0.002, p = 0.003; β = - 0.007, 95% CI: 0.011 to - 0.003, p < 0.001, respectively) (Table 3).

**TABLE 3:** Unadjusted generalized linear mixed models assessing the association of HDP and PE with neonatal crSO_2_/cFTOE, without adjusted for confounders.

Measurement	*β*	SE	t	*P*	Lower limit	Upper limit
crSO_2_						
HDP	0.520	0.1663	3.125	0.002	0.194	0.846
PE	0.663	0.1827	3.631	<0.001	0.305	1.022
cFTOE						
HDP	−0.005	0.0018	−2.989	0.003	−0.009	−0.002
PE	−0.007	0.0019	−3.523	<0.001	−0.011	−0.003

Abbreviations: HDP, hypertensive disorders of pregnancy; PE, preeclampsia; crSO_2_, cerebral regional tissue oxygen saturation; cFTOE, cerebral fractional tissue oxygen extraction; *β,* regression coefficient; SE, standard error.

After adjusting for confounders, the associations remained significant. HDP and PE were independently associated with higher crSO_2_ (β = 0.472, 95% CI: 0.122 to 0.823, p = 0.008; β = 0.625, 95% CI: 0.234 to 1.017, p = 0.002, respectively) and lower cFTOE (β = - 0.004, 95% CI: 0.008 to −0.001, p = 0.027; β =−0.006, 95% CI: 0.010 to - 0.002, p = 0.006, respectively) (Table 4).

**TABLE 4:** Generalized linear mixed models assessing the association of HDP and PE with neonatal crSO_2_/cFTOE, adjusted for confounders*.

Measurement	β	SE	t	*P*	Lower limit	Upper limit
crSO_2_						
HDP	0.472	0.1788	2.642	0.008	0.122	0.823
PE	0.625	0.1998	3.131	0.002	0.234	1.017
cFTOE						
HDP	−0.004	0.0019	−2.216	0.027	−0.008	−0.001
PE	−0.006	0.0022	−2.776	0.006	−0.010	−0.002

*adjust for GA and BW. The Variance inflation factors of GA and BW were 2.804 and 2.804, respectively. HDP, hypertensive disorders of pregnancy; PE, preeclampsia; crSO2, cerebral regional tissue oxygen saturation; cFTOE, cerebral fractional tissue oxygen extraction; GA, gestational age; BW, birth weight.

### 3.4 Partial mediation analysis

**FIGURE 4:**
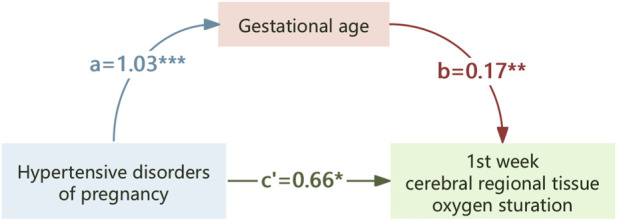
The mediating effect of gestational age on the association of hypertensive disorders of pregnancy on crSO_2_. *,p < 0.05; **,p < 0.01; ***,p < 0.001.

**FIGURE 5:**
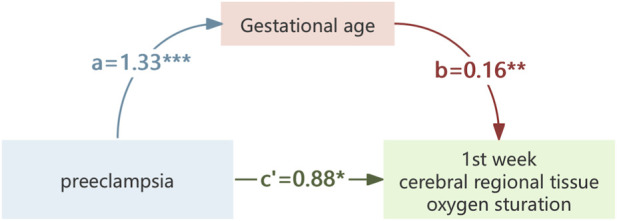
The mediating effect of gestational age on the association of preeclampsia on crSO_2_. *,p < 0.05; **,p < 0.01; ***,p < 0.001.

Mediation analyses were conducted to assess the role of GA in the associations between HDP or PE and crSO_2_ during the first week of life. As shown in Table 5, preterm birth may be a mediating association of HDP or PE on crSO_2_ in offspring. Mediation analysis suggested that GA exerts a partial mediating role in the association between HDP or PE with crSO_2_. Specifically, HDP could influence crSO_2_ partially by affecting GA levels (Figure 4), and PE could also exert a partial effect on crSO_2_ via GA (Figure 5).

**TABLE 5:** Mediation analysis of gestational age in the associations between HDP/PE and neonatal crSO_2_.

Exposure	Effect type	Effect size (β)	95% CI	Proportion mediated (%)	*P*
HDP					
	Total effect	0.90	(0.25, 1.72)	100.0	<0.001
	Direct effect	0.72	(0.10, 1.47)	79.66	0.040
	Indirect effect	0.18	(0.06, 0.28)	20.34	<0.001
PE					
	Total effect	1.17	(0.25, 1.72)	100.0	<0.001
	Direct effect	0.96	(0.22, 1.82)	82.15	<0.001
	Indirect effect	0.21	(0.06, 0.33)	17.85	<0.001

Adjust, fetal growth restriction, advanced maternal age at first delivery, hypoproteinemia, hypothyroidism or hyperthyroidism, gestational diabetes, intrahepatic cholestasis of pregnancy, MgSO_4_. HDP, hypertensive disorders of pregnancy; crSO_2_, cerebral regional tissue oxygen saturation; GA, gestational age; BW, birth weight.

## 4 Discussion

The neonatal brain appears to be highly sensitive to oxygen fluctuations due to its elevated metabolic demand, both hypoxia and hyperoxia can affect it (Baik-Schneditz et al., 2021), and crSO_2_ serves as a reliable indicator of cerebral function in neonates (Ng et al., 2020; Vesoulis et al., 2021b). This study utilized repeated NIRS measurements in a large number of neonates (n = 928) to evaluate how HDP correlation cerebral oxygenation in offspring. Our findings demonstrated that HDP may significantly influenced neonatal cerebral oxygen during the first 2 weeks of life, characterized by elevated crSO_2_ levels and reduced cFTOE. However, these differences diminished thereafter. Notably, preterm birth (represented by GA) could partly mediated the relationship between HDP and crSO_2_, accounting for 20.34% of the observed association.

Subgroup analysis further revealed consistent results among neonates born to mothers with PE, a prominent subtype of HDP. These observations contribute to our understanding of the potential neurological impact of intrauterine HDP exposure and highlight the mediating role of prematurity in altering neonatal brain oxygenation.

In this study, the higher crSO_2_ and lower cFTOE observed in neonates with HDP and PE may be associated with lower GA and the resultant decline in cerebral blood flow autoregulation. Neonatal cerebral vessels autonomously regulate cerebral blood flow within a certain range of peripheral circulation fluctuations to stabilize cerebral blood flow and oxygen supply (Demel et al., 2015). The lower the gestational age, the less developed the capacity for cerebral autoregulation tends to be (Conti-Ramsden et al., 2024; Bouyssi-Kobar et al., 2018). A study investigated crSO_2_ in 70 preterm infants. The results demonstrated that crSO_2_ levels decreased as GA increased. Specifically, the crSO_2_ values in infants born at 24 and 28 weeks of gestation were 76.4% and 74.6%, respectively, measured 24 h after birth. In contrast, cFTOE showed an increasing trend with advancing GA (Mohamed et al., 2021). The difference in crSO_2_ between the HDP and PE group with the control group gradually decreased after 2 weeks, which may be attributed to the progressive improvement of cerebral blood flow autoregulatory function (Bouyssi-Kobar et al., 2018).

In this study, we adjusted for maternal medication use, including antihypertensive agents and magnesium sulfate, in our regression models. This enhanced the reliability of our findings by reducing potential confounding from pharmacologic exposure. Although earlier studies have suggested that maternal medications such as magnesium sulfate and beta-blockers may affect neonatal hemodynamics (Shokry et al., 2010), our results indicate that the changes in crSO_2_ and cFTOE were independently associated with HDP and PE.

HDP and PE are well-known risk factors for preterm birth, and the severity of maternal hypertension correlates positively with the risk of medically indicated preterm delivery (Conti-Ramsden et al., 2024). Previous studies involving preterm infants have indicated that gestational age (GA) significantly correlation cerebral regional oxygen saturation (crSO_2_); however, the direction and nature of this association vary across studies. For instance, Mohamed et al. found that in preterm infants born at ≤34 weeks’ GA, crSO_2_ levels decreased as gestational age increased, suggesting that relatively mature preterm infants had lower crSO_2_ but greater cerebral oxygen extraction capacity (Mohamed et al., 2021). In contrast, another study observed that infants with lower gestational age experienced more frequent episodes of reduced cerebral oxygen saturation (Mohamed et al., 2025). These differing results highlight the complexity of the relationship between gestational maturity and cerebral oxygenation dynamics.

In our study, mediation analysis was utilized to clarify how HDP association neonatal crSO_2_ levels during early life. We found that HDP had both a direct association (0.72, accounting for 79.66% of the total association) and an indirect association mediated by GA (0.18, accounting for 20.34%) on neonatal crSO_2_ in the first week of life. The subgroup analysis of neonates born to mothers with PE revealed similar results. These findings collectively indicate that the association of HDP on neonatal crSO_2_ involves direct physiological disturbances related to maternal hypertension and indirect association mediated by preterm birth. Future studies exploring the specific mechanisms linking GA and cerebral oxygenation in neonates exposed to HDP are therefore warranted.

A major strength of our study is the use of repeated crSO_2_ monitoring throughout the neonatal period in a large cohort, enabling us to clearly characterize both the magnitude and duration of HDP’s association on neonatal cerebral oxygenation. However, due to technical constraints, NIRS cannot directly measure local tissue oxygen saturation but instead provides an indirect estimation by quantifying the oxygenated and deoxygenated hemoglobin concentrations. Furthermore, prolonged continuous monitoring is limited by the fragility of neonatal skin, which restricts the assessment of fine-grained temporal changes in cerebral oxygenation. Additionally, as this case–control study included only NICU-admitted infants who underwent NIRS monitoring, potential sampling bias cannot be excluded. Both HDP and non-HDP groups were drawn from hospitalized populations with similar morbidity risks, which may limit the generalizability of the findings and obscure the independent effects of gestational age and NICU admission indications. Future studies using population-based samples that include both hospitalized and non-hospitalized neonates are needed to minimize selection bias and clarify the effects of HDP and gestational age on cerebral oxygenation.

Furthermore, it should be noted that crSO_2_ measured by NIRS represents an indirect and relative estimate of cerebral oxygenation rather than a direct quantification of brain tissue oxygen levels. The NIRS signal is affected by multiple factors such as scalp thickness, sensor positioning, extracerebral tissue interference, and device-specific calibration algorithms. Consequently, while NIRS provides valuable trend information about cerebral oxygenation and tissue oxygen extraction, its absolute values should be interpreted with caution. Future studies incorporating multimodal assessments—such as Doppler ultrasonography or MRI-based perfusion imaging—and population-based sampling will be needed to validate and extend these findings.

Another limitation of this study is the data loss at later follow-up points. Because cerebral oxygenation data for the third and fourth postnatal weeks were incomplete, GLMM analyses were limited to the first 2 weeks, and the group-by-time interaction term was not included to ensure model stability. The analysis therefore focused on the effect of maternal HDP in the early postnatal period rather than on longitudinal trends. Future studies with complete follow-up data are needed to better assess temporal patterns and interaction effects.

Moreover, this study employed a non-matched design. Although we adjusted for known confounders through statistical models, residual confounding from unmeasured factors may still persist. Additionally, compared to a matched design, a non-matched design may have slightly lower statistical power with the same sample size.

Additionally, the cranial ultrasound findings were recorded using a grading-based system in clinical practice. As suggested by Leviton and Paneth (2004), a descriptive classification focusing on the location and nature of the hemorrhage would provide a more precise depiction of brain injury. However, reclassification was not feasible in this retrospective analysis due to the lack of raw imaging data.

Our results indicated significant differences in crSO_2_ and cFTOE between neonates in the HDP and PE groups compared with controls during the first 2 weeks of life, but these differences diminished thereafter. Further research is necessary to confirm and extend these findings. Currently, the relationship between early postnatal cerebral oxygenation changes and long-term neurological outcomes remains uncertain. Therefore, longitudinal follow-up studies are urgently needed to determine whether early cerebral oximetry profiles can serve as predictive markers of adverse outcomes.

If validated in future prospective cohorts, early postnatal crSO_2_ abnormalities could identifying neonates at elevated neurological risk due to maternal HDP. This may open avenues for developing early intervention strategies, such as individualized monitoring or cerebral perfusion–targeted therapies, aimed at improving neurodevelopmental outcomes in this high-risk population.

## 5 Conclusion

In this case-control study, maternal HDP was associated with increased crSO_2_ and reduced cFTOE in neonates during the first 2 weeks of life. Gestational age was identified as a partial mediator of this relationship. These findings provide evidence of altered cerebral oxygenation patterns in neonates exposed to HDP and support further investigation into the clinical relevance and long-term implications of early postnatal crSO_2_ changes in this population.

## Data Availability

The datasets presented in this article are not readily available because the dataset is restricted to academic research only, requiring signed data-use agreements. Redistribution is prohibited, and all analyses must be anonymized. Access must be approval to project review. Requests to access the datasets should be directed to Yao Zhang, zhangyaoaoo@qq.com.
